# Emerging Infections Program—20 Years of Achievements and Future Prospects

**DOI:** 10.3201/eid2109.150564

**Published:** 2015-09

**Authors:** Ruth Lynfield, William Schaffner

**Affiliations:** Minnesota Department of Health, St. Paul, Minnesota, USA (R. Lynfield);; Vanderbilt University School of Medicine, Nashville, Tennessee, USA (W. Schaffner)

**Keywords:** Emerging Infections Program, EIP, surveillance, public health, achievements, future prospects

“Disease-causing microbes have threatened human health for centuries. The Institute of Medicine’s Committee on Emerging Microbial Threats to Health believes that this threat will continue and may even intensify in coming years” (*1*). Thus begins the Institute of Medicine’s 1992 Report on Emerging Infections. The Institute of Medicine indicated that “emergence may be due to the introduction of a new agent, to the recognition of an existing disease that has gone undetected, or to a change in the environment that provides an epidemiologic bridge.” The recommendations encompassed both the ability to detect (surveillance) and respond to emerging infections. These recommendations laid the groundwork for establishment of the Emerging Infections Program (EIP).

This issue of Emerging Infectious Diseases marks the 20th anniversary of the EIP. Sponsored and organized by the Centers for Disease Control and Prevention (CDC), the EIP is a multifaceted collaboration of CDC with 10 state health departments and their academic partners, with the goal of conducting a portfolio of work that can be characterized as enhanced public health surveillance and applied research to detect, prevent, and control emerging infectious diseases. Collaboration derives from the Latin word “collaboratus,” meaning to labor together. The collaboration has been profound and successful, with marked commitment, creativity, and passion contributed by all participants.

This special issue incorporates a Festschrift for the EIP, celebrating the accomplishments of this distinctive enterprise over the past 2 decades. The first article of the series uses a tree metaphor to describe the history of the EIP over the past 20 years and discusses future directions for the network. The following article provides a state-based perspective, which includes the enhancement of public health infrastructure and the development of new academic and public health partnerships. Another article describes the considerable training and teaching activities undertaken by EIP investigators. Although training was among the consortium’s explicit goals when EIP was initiated, its funding has been evanescent, thus requiring commitment and imaginative flexibility to create training opportunities in the context of active investigations. However, EIP investigators have derived great pleasure in training the next generation of public health epidemiologists, and this has yielded dividends for mentees, mentors and public health.

These initial articles are followed by a series of reviews that summarize and assess core EIP areas and some related noteworthy projects. The network has successfully established population-based surveillance for many pathogens of public health importance and has been able to provide insights into risk factors for disease, and characterization of pathogens. EIP data have been used to inform public health recommendations for the prevention and control of multiple infectious diseases and to evaluate public health interventions. Not resting on their laurels, the authors of these articles also look to future challenges, including those directly related to the infections, as well as others imposed by health inequities and changes in technology. A series of original research contributions by EIP investigators and their collaborators follows the reviews.

The scientific work of the EIP is directed through a genuinely collaborative steering committee comprised of lead investigators from all sites in the field, as well as CDC. It is co-chaired by a CDC investigator and a site senior investigator. Priority-setting discussions are open and genial, informed equally by national views and local perspectives. Formal votes are rare; consensus building is the norm. The participants have longevity; many have been with the program since its inception and have nurtured it through 2 decades of administrative, fiscal, and scientific labyrinths. As such, the participants have become true partners and value the mutual trust, sense of harmony, and friendships that have flourished over the years. These qualities, along with a shared commitment to science-based public health practice, have led to the success of the EIP and bode well as the network looks forward to tackling the next generation of emerging issues of public health importance.
